# Is Brain-Derived Neurotropic Factor Methylation Involved in the Association Between Prenatal Stress and Maternal Postnatal Anxiety During the COVID-19 Pandemic?

**DOI:** 10.3389/fpsyt.2022.950455

**Published:** 2022-07-14

**Authors:** Livio Provenzi, Marco Villa, Fabiana Mambretti, Andrea Citterio, Serena Grumi, Emanuela Bertazzoli, Giacomo Biasucci, Lidia Decembrino, Barbara Gardella, Roberta Giacchero, Maria Luisa Magnani, Renata Nacinovich, Camilla Pisoni, Federico Prefumo, Simona Orcesi, Barbara Scelsa, Roberto Giorda, Renato Borgatti

**Affiliations:** ^1^Department of Brain and Behavioral Sciences, University of Pavia, Pavia, Italy; ^2^Child Neurology and Psychiatry Unit, IRCCS Mondino Foundation, Pavia, Italy; ^3^Scientific Institute IRCCS E. Medea, Bosisio Parini, Italy; ^4^ASST Lodi, Lodi, Italy; ^5^Guglielmo da Saliceto Hospital, Piacenza, Italy; ^6^ASST Pavia, Pavia, Italy; ^7^Fondazione IRCCS Policlinico San Matteo, Pavia, Italy; ^8^ASST Monza, Monza, Italy; ^9^Department of Medicine and Surgery, Università Bicocca, Milan, Italy; ^10^ASST Spedali Civili, Brescia, Italy; ^11^Division of Obstetrics and Gynecology, Department of Clinical and Experimental Sciences, University of Brescia, Brescia, Italy; ^12^ASST Sacco Fatebenefratelli, Milan, Italy

**Keywords:** anxiety, *BDNF*, COVID-19, methylation, epigenetics, pandemic, pregnancy, stress

## Abstract

**Background:**

The COVID-19 pandemic is a collective trauma that may expose susceptible individuals to high levels of stress. Pregnant women represent a high-risk population, considering that pregnancy is a period of heightened neuroplasticity and susceptibility to stress through epigenetic mechanisms. Previous studies showed that the methylation status of the *BDNF* gene is linked with prenatal stress exposure. The goals of this study were (a) to assess the association between pandemic-related stress and postnatal anxiety and (b) to investigate the potential role of maternal *BDNF* methylation as a significant mediator of this association.

**Methods:**

In the present study, we report data on the association among pandemic-related stress during pregnancy, maternal *BDNF* methylation, and postnatal anxiety symptoms. Pandemic-related stress and postnatal anxiety were assessed through self-report instruments. *BDNF* methylation was estimated in 11 CpG sites in DNA from mothers’ buccal cells. Complete data were available from 108 mothers.

**Results:**

Results showed that pandemic-related stress was associated with an increased risk of postnatal anxiety, *r* = 0.20, *p* < 0.05. CpG-specific *BDNF* methylation was significantly associated with both prenatal pandemic-related stress, *r* = 0.21, *p* < 0.05, and postnatal maternal anxious symptoms, *r* = 0.25, *p* = 0.01. Moreover, a complete mediation by the *BDNF* CpG6 methylation emerged between pandemic-related stress during pregnancy and postnatal maternal anxiety, ACME = 0.66, *p* < 0.05.

**Conclusion:**

These findings suggest that BDNF epigenetic regulation by pandemic-related stress might contribute to increase the risk of anxiety in mothers. Policymakers should prioritize the promotion of health and wellbeing in pregnant women and mothers during the present healthcare emergency.

## Introduction

Pregnancy is a period of heightened neuroplasticity for women (1). Changes in brain connectivity and neuroendocrine regulation are meant to facilitate the transition to motherhood and to prepare the women to develop appropriate caregiving skills and attachment sensitivity to the newborn soon after delivery (2, 3). Nonetheless, this same heightened neuroplasticity may also result in increased susceptibility to adverse conditions and stressful exposures during pregnancy (4–6). The consequences of prenatal stress may be deleterious for women, and they may set the stage for a greater risk of developing anxious symptoms, which may impair not only maternal mental health but also the early establishment of an intimate and reciprocally satisfying relationship with the infant. Recent research suggests that environmental stress may alter epigenetic mechanisms—such as DNA methylation—at specific sites of genes involved in stress reactivity and regulation.

The COVID-19 pandemic is an unprecedented healthcare emergency and at the same time a prolonged and unpredictable collective trauma that has dramatically affected every domain of our life. The fear of contagion, the partial knowledge of the virus and its implications, together with the lockdown limitations that were key to the success of mitigation and containment strategies are sources of psychological distress that should not be underestimated in at-risk individuals. A recent meta-analytic study suggested that during the pandemic anxiety—rather than depressive—symptomatology may be heightened in pregnant and postpartum women (7). In the present study, we report on the association between pandemic-related stress experienced by women during pregnancy and maternal anxious symptoms after delivery. Moreover, we highlight the role played by the DNA methylation of a specific stress-related target gene—namely, the *BDNF* gene—in mediating this relationship.

### Neuroplasticity and Stress Susceptibility During Pregnancy

During pregnancy, the neurobiology of mothers undergoes dramatic changes that involve regulatory processes occurring at the level of the central nervous system and different neuroendocrine axes (1). A great variety of intertwined functional and structural changes occurs in the female brain throughout pregnancy and may continue during the postpartum period. These neurobiological adaptations are meant to be largely informed by neuroendocrine and environmental triggers (8, 9). Specific brain areas in which variations in brain volume occur involve the medial preoptic area (mPOA) and the hippocampus (10, 11), brain areas that have well-known associations with the emergence of specific caregiving behaviors in both animal models and humans (12, 13). Mechanisms underlining the restructuring of the maternal brain across pregnancy involve neurogenesis, synaptic remodeling, and reshaping of dendrites (1, 14–16).

Such a general reconfiguration of maternal neurobiology has relevant implications for the susceptibility of pregnant women to stressful exposures. Indeed, as pregnancy is a time windows of increased interaction between genes and environmental exposures, it is also a critical period for regulation triggered by adverse and stressful conditions (17). Heightened risk of stress-related risk conditions has been highlighted in women exposed to adverse events during pregnancy (18, 19). Rates of postpartum anxiety range from 10 to 17% (20, 21) and anxious symptoms reported by mothers after delivery have often precursors in stress experiences during pregnancy (22). A history of stress and adverse conditions during pregnancy is one of the most significant antecedents of postnatal anxiety in mothers (23). Timely identification of prenatal risk and postnatal signs of maternal anxiety is crucial in clinical settings as untreated maternal anxiety may have a plethora of consequences for both women’s later psychological adjustment to motherhood and child developmental trajectories (5, 24–28).

### The Brain-Derived Neurotropic Factor Gene: An Epigenetic Target for Stress Exposure and Psychiatric Risk

Among the mechanisms involved in setting the risk for stress susceptibility during pregnancy, the epigenetic regulation of specific stress-related genes has been recently reviewed and confirmed (29). Behavioral epigenetics refers to alterations of the DNA function that are highly malleable in response to environmental exposures, that do not involve mutations of the dinucleotide sequence, and that can affect gene expression and protein synthesis (30). In other words, whereas the genome consists of the genetic information contained in the DNA that informs gene transcription and expression, the epigenome defines which genes of this repertoire are actually expressed (31). DNA methylation is by far the most investigated epigenetic mechanism in animal and human neurobehavioral studies. It occurs when a methyl group binds to specific 5′-cytosine guanine-3′ dinucleotides (i.e., CpG sites) and may contribute to reducing gene expression (i.e., gene silencing) (32). Adverse exposures occurring during specific temporal window of heightened neuroplasticity and susceptibility to stress may be especially capable of leaving epigenetic marks capable of contributing to the dysregulation of key physiologic, neuroendocrine, and neurobehavioral systems (33). Moreover, DNA methylation is of specific concern when it occurs at the level of stress-related genes that are known for their implications in behavioral, cognitive, and socio-emotional development as well as in the promotion of physical and mental health (34, 35).

The brain-derived neurotropic factor (*BDNF*) gene may be a specific target gene of interest that has shown to be susceptible to epigenetic regulation following stressful exposures (36) and to be significantly associated with increased risk of psychiatric disorders, including anxious symptomatology (37). *BDNF* is a member of the neurotrophic growth factor family. It contains 11 exons in humans, nine of which include promoters that regulate its expression (38, 39). A large variety of cells express the *BDNF* molecule in different tissues using different splice sites, leading to the formation of numerous *BDNF* transcripts variants (40). It plays key functions in the regulation of proliferation, growth, maintenance, and survival of specific target neurons during pregnancy and in postnatal life (41, 42). Like other neurotrophins, *BDNF* is essential for the outgrowth and activity-dependent neuroplastic remodeling that occurs during pregnancy (43, 44).

Notably, the *BDNF* gene is susceptible to epigenetic regulation by environmental stimulations, and this may be especially true during time windows of heightened neuroplasticity like pregnancy (45, 46). Environmental challenges and threats occurring during pregnancy may affect *BDNF* methylation profiles both in the brain and in peripheral tissues, such as blood and buccal cells (46, 47). Increased *BDNF* methylation has been documented in response to adverse life conditions in central and peripheral tissues of both animal models and humans (48, 49) and similar trends in *BDNF* methylation have been reported between peripheral and central assessments (37). CpG sites located in different exons may show environmentally regulated changes in their methylation status; nonetheless, the specific CpG sites and loci of epigenetic regulation of the *BDNF* gene by environmental stress exposures only partially overlap among different studies (37, 40). Higher stress-related serum cortisol has been linked with concurrent reduction in *BDNF* serum expression during the second trimester of pregnancy (50).

Animal models suggest that variations in *BDNF* expression may be mirrored in impairments of learning, memory, and social behavior, including anxiety-like traits (51). Associations with stress-related mood disorders and anxiety have been also reported in humans assessing *BDNF* methylation in peripheral tissues, such as blood and saliva (36, 52–55). Notably, previous research mainly focused on the effects of prenatal stress on the regulation of the *BDNF* methylation status in the offspring, highlighting statistically significant positive associations (47, 56, 57). Despite the fact that stress-related increases in glucocorticoids during pregnancy have been found to be associated with a lower synthesis of maternal *BDNF* (58), little is known about the effects of stress during pregnancy on the epigenetic regulation of maternal *BDNF* and on the subsequent risk for mental health, such as anxiety symptomatology.

### The Present Study

The COVID-19 pandemic is an unprecedented healthcare emergency that is challenging all the domains of our daily life. Its rapid spread and the lack of complete knowledge about the virus resulted in the employment of population-based behavioral strategies to contain and manage the contagion. As these strategies resulted in prolonged and repeated lockdown periods, psychological stress emerged as a non-negligible side effect of the pandemic on a global scale. As the exposure to stress is of particular concern during time windows of heightened neuroplasticity, we wondered whether and how this collective trauma was affecting the health of women and infants. As such, we launched the Measuring the Outcomes of Maternal COVID-19-related Prenatal Exposure (MOM-COPE) research project in April 2020. The MOM-COPE project is a multi-centric and prospective study that involves ten neonatal units in Northern Italy and that includes the collection of self-report, behavioral, and epigenetic correlates of pandemic-related stress during pregnancy and further health-related and development outcomes from birth to 12-month-age of the infant (59). In the present study, we report on the association among pandemic-related stress during pregnancy, maternal BDNF methylation, and postnatal anxiety symptoms. Our first goal was to assess the association between pandemic-related stress and postnatal anxiety. Based on the literature reviewed above, we hypothesized a positive and significant relationship, with mothers reporting higher prenatal stress showing also the greatest elevations in postnatal anxious symptoms. Our second goal was to assess the role of maternal BDNF methylation as a significant mediator of this association. As suggested by previous research in animal models and humans, we hypothesized that (a) higher prenatal pandemic-related stress would be associated with increased methylation of the BDNF gene and (b) such an altered epigenetic status would associate with greater reports of anxiety after delivery. As previous research did not univocally highlight specific candidate CpG sites, we explored this association by focusing on a CpG-rich locus in the promoter region of the BDNF gene.

## Materials and Methods

### Participants

The MOM-COPE is a prospective and multi-centric cohort study that involves ten neonatal units in Northern Italy and is aimed at highlighting the behavioral and epigenetic consequences of prenatal pandemic-related stress during the COVID-19 emergency for maternal health and infants’ development. The fully detailed description of this project is reported elsewhere (59). Here we report on a sample of 108 mothers with complete prenatal (T_0_) and neonatal (T_1_) data between May 2020 and February 2021. Mothers were included if at least 18-year-old, in the absence of prenatal and perinatal diseases or injuries, if they delivered at term (i.e., from 37 + 0 to 41 + 6 weeks of gestation), and if they were negative for COVID-19 at delivery. Mothers were not considered eligible to the study in presence of any maternal or infants’ comorbidity.

### Ethics

The study was approved on April, 8th 2020 by the Ethics Committees (protocol ID 20200037366) of the project lead institution (IRCCS Mondino Foundation, Pavia, Italy) and the participating hospitals. All the procedures were performed in accordance with the 2018 Declaration of Helsinki for studies conducted with human participants. All mothers provided informed consent to participate to the study.

### Procedures

Mothers were first contacted at antepartum classes or immediately following the postpartum period. Socio-demographic and neonatal data were obtained from medical records. Within 48 h from delivery, the mothers filled in a first set of questionnaires to provide retrospective quantitative measures of prenatal COVID-19-related stress and present anxiety symptoms. Between 6 and 24 h, buccal cells were obtained from mothers to assess *BDNF* methylation.

### Measures

#### Sample Characteristics

Mothers self-reported socio-demographic characteristics (i.e., age, educational level, and occupational status), pandemic-related stress during pregnancy, and present anxious symptoms. Neonatal characteristics (i.e., gestational age, birth weight, head circumference, neonatal length, Apgar at minute 1, breastfeeding at birth, and mode of delivery) were collected from medical records.

#### Questionnaires

For pandemic-related stress, an *ad hoc* questionnaire was developed to target dimensions of stress specifically related to the present COVID-19 healthcare emergency (Items are reported in Table 1); a mean score was obtained, ranging from 1 (low) to 5 (high). Anxious symptoms were assessed using the State-Trait Anxiety Inventory (STAI-Y) (60), a well-validated questionnaire that includes 20 items and provides a global score ranging from 20 (low) to 80 (high). A STAI-Y score above 40 is usually index of clinically relevant elevations in anxious symptoms. Mothers were considered eligible to the study only if negative to SARS-CoV-2. Nonetheless, PCR testing could not exclude direct or indirect exposures to the virus that thus were explored with *ad hoc* items indicating whether they had symptoms reminiscent of COVID-19 in the previous months, whether their relatives or significant others were positive to the virus, as well as whether they were hospitalized in intensive care units and/or eventually died with COVID-19. The physical direct/indirect exposure to the COVID-19 virus was dichotomized as 0 (no exposure) and 1 (at least one direct or indirect exposure).

**TABLE 1:** Pandemic-related stress questionnaire.

	Pandemic-related stress (Response: 5-point Likert scale)
	During pregnancy…
1	How much worried were you about the risk of COVID-19 infection?
2	How much did you feel that your pregnancy was at risk due to COVID-19 pandemic?
3	How much did you fear for your health?
4	How much did you fear for your baby’s health?
5	How much did you feel that you were losing confidence in your health?
6	How much did you feel you had lost faith in medicine?

#### Brain-Derived Neurotropic Factor Methylation

**FIGURE 1:**
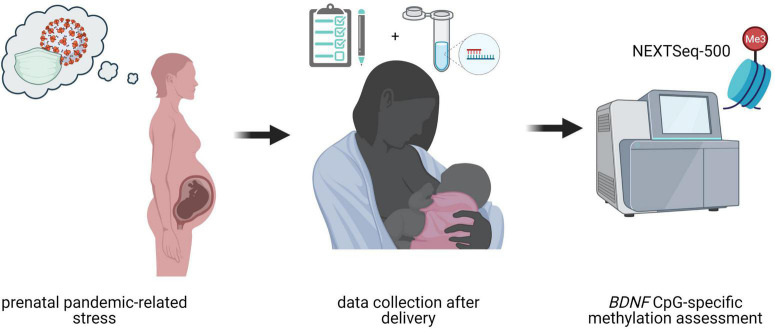
Methodology of the study.

Maternal buccal cells samples were collected using the OraCollect kit OC-175 (DNA Genotek, Ottawa, Canada) between 6 and 24 h from delivery. Methylation assessment was conducted according to previous validated procedures from this lab (61, 62). The genomic DNA was extracted following manufacturer’s protocols and its quality was assessed using a Qubit fluorimeter (Invitrogen, Thermo Fisher Scientific, Waltham, Massachusetts, United States). The methylation status of 11 CpG sites in the *BDNF* gene promoter region (chr11: 27,723,096–27,723,219; see Table 2 for CpG-specific positions) was assessed by PCR amplification of bisulfite-treated DNA followed by Next Generation Sequencing (NGS) on a NEXTSeq-500 (Illumina, San Diego, California, United States). The region was selected based on previous research on the association of *BDNF* methylation with maternal mental health and stress (46). Figure 1 illustrates the study methodology.

**TABLE 2:** Positions of the selected *BDNF* CpG sites human genome assembly GRCh37 (hg19).

CpG site #	Position
1	Chr11: 27,723,218–27,723,219
2	Chr11: 27,723,214–27,723,215
3	Chr11: 27,723,203–27,723,204
4	Chr11: 27,723,190–27,723,191
5	Chr11: 27,723,161–27,723,162
6	Chr11: 27,723,159–27,723,160
7	Chr11: 27,723,143–27,723,144
8	Chr11: 27,723,137–27,723,138
9	Chr11: 27,723,128–27,723,129
10	Chr11: 27,723,125–27,723,126
11	Chr11: 27,723,095–27,723,096

#### Plan of Analysis

Variables of interest (pandemic-related stress, anxious symptoms, and CpG-specific *BDNF*% methylation) were first tested for normal distribution. Kurtosis and asymmetry were within the ± 2 range and no outliers (values over ± 3 standard deviations from the mean) were detected. The presence of significant differences in variables of interest by exposure to the COVID-19 virus was tested with independent-sample *t*-tests. Separate Pearson’s bivariate correlations were used to assess the presence of significant associations among pandemic-related stress during pregnancy, maternal anxious symptoms after delivery, and CpG-specific *BDNF*% methylation. Multiple-testing bias was checked using the Benjamini-Hochberg procedure, *q* < 0.05. CpG-specific *BDNF*% methylation values for which a significant association emerged with both pandemic-related stress and anxious symptoms were subsequently tested in a mediation model to assess their role as significant mediators of the relationship between pandemic-related stress and maternal anxious symptoms. The model was tested using R (version 4.0.0) (63) mediation package (64). A *post hoc* power analysis setting medium size effect, *alpha* = 0.05, and sample size 108 revealed an adequate power of 0.89. The statistical analyses were carried setting *p* < 0.05.

## Results

The socio-demographic descriptive statistics for the sample are reported in Table 3. The Cronbach alpha for the pandemic-related stress questionnaire was 0.83, suggesting a satisfactory internal consistency. All items loaded on a single factor solution with loadings above 0.72. Thirty-four mothers (32%) reported STAI-Y scores higher than the clinical cut-off. No statistically significant differences emerged for pandemic-related stress during pregnancy, maternal anxious symptoms post-delivery, and *BDNF* promoter region CpG-specific% methylation values between individuals with or without any direct/indirect exposure to the COVID-19 virus (Table 4).

**TABLE 3:** Descriptive statistics.

	Min	Max	Mean	SD
Gestational age (weeks)	37.00	42.00	39.71	1.05
Birth weight (grams)	2430.00	4345.00	3342.88	413.82
Apgar at minute 1	6.00	10.00	9.18	0.69
Maternal educational level (years of study)	5.00	23.00	14.44	3.57
			N	%
Infant’s sex (females)			55	50.9
Delivery (eutocic)			69	63.9
Maternal occupational status (employed)			95	88.0

**TABLE 4:** Comparison between mothers with and without any direct or indirect exposure to the COVID-19 during pregnancy for variables of interest.

	Exposure to the COVID-19 virus					
	No (*n* = 59)			Yes (*n* = 49)		
	Mean	SD	ES	Mean	SD	ES
Pandemic-related stress	2.34	0.62	0.08	2.54	0.70	0.10
Anxious symptoms	35.68	9.86	1.28	34.61	9.58	1.37
*BDNF* CpG-specific% methylation						
CpG 1	1.31	0.61	0.08	1.34	0.48	0.07
CpG 2	0.46	0.22	0.03	0.54	0.25	0.04
CpG 3	0.54	0.24	0.03	0.58	0.28	0.04
CpG 4	0.32	0.14	0.02	0.37	0.18	0.03
CpG 5	0.69	0.35	0.05	0.81	0.64	0.09
CpG 6	0.51	0.24	0.03	0.52	0.19	0.03
CpG 7	0.57	0.27	0.04	0.57	0.27	0.04
CpG 8	0.66	0.31	0.04	0.67	0.29	0.04
CpG 9	0.69	0.33	0.04	0.69	0.30	0.04
CpG 10	0.93	0.36	0.05	0.88	0.33	0.05
CpG 11	0.73	0.36	0.05	0.74	0.27	0.04

**FIGURE 2:**
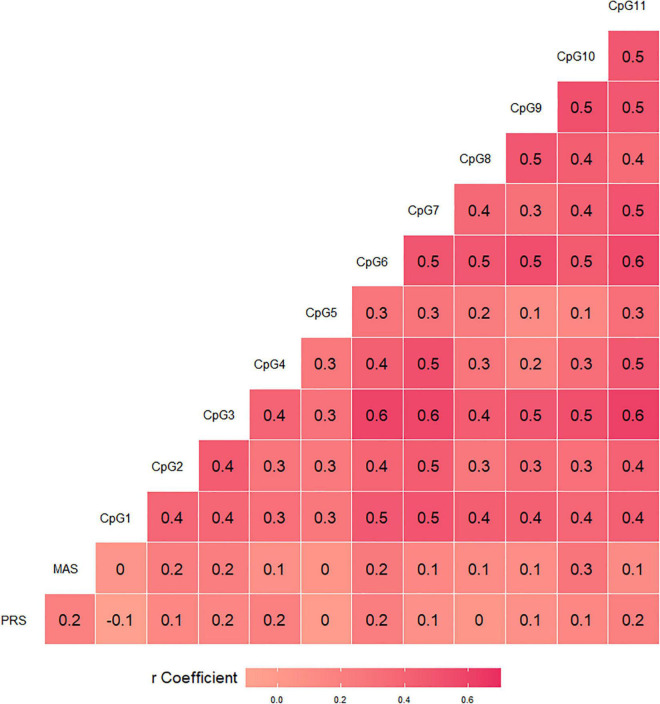
Bivariate correlations of *BDNF* CpG-specific percentage methylation with pandemic-related stress (PRS) during pregnancy and postnatal maternal anxious symptoms (MAS).

A significant correlation emerged between pandemic-related stress and postnatal maternal anxious symptoms, *r* = 0.20, *p* < 0.05. The associations of *BDNF* CpG-specific% methylation values with pandemic-related stress during pregnancy and postnatal maternal anxious symptoms are reported in Figure 2. Pandemic-related stress was significantly correlated with CpG sites 4 (*r* = 0.20, *p* = 0.037), 6 (*r* = 0.21, *p* = 0.027), and 11 (*r* = 0.20, *p* = 0.040). Anxious symptoms were significantly correlated with CpG sites 2 (*r* = 0.21, *p* = 0.027), 3 (*r* = 0.21, *p* = 0.026), 6 (*r* = 0.25, *p* = 0.011), and 10 (*r* = 0.28, *p* = 0.003). All significant associations survived Benjamini-Hochberg check.

**FIGURE 3:**
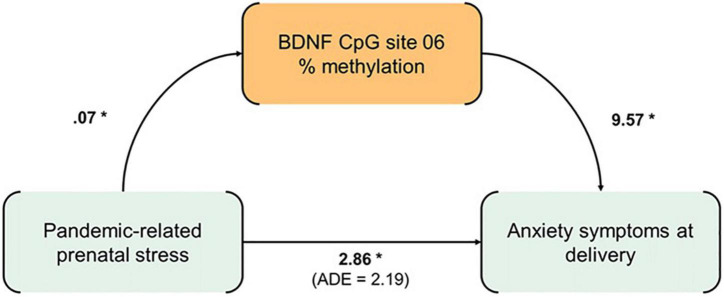
Mediation model. *p < 0.05.

As *BDNF* CpG6 showed significant associations with both prenatal pandemic-related stress and postnatal maternal anxious symptoms, the methylation value at this CpG site was tested in the mediation model (Figure 3). A complete mediation by *BDNF* CpG6% methylation emerged (ACME = 0.66, 95% C.I. (0.00, 1.83), *p* < 0.05; ADE = 2.19, 95% C.I. (–0.55, 4.89), *p* > 0.10.

## Discussion

In this study, we were interested in investigating the association between prenatal pandemic-related stress experienced by women during the COVID-19 healthcare emergency and the levels of anxious symptoms reported after delivery. Moreover, we wanted to assess the role played by the methylation status of the *BDNF* promoter region in mediating this association, as previous research suggested that this gene might be susceptible to epigenetic regulation by adverse conditions occurring during pregnancy. Our findings are consistent with previous literature, suggesting that increased methylation of this gene may be involved in setting the risk for heightened anxious symptoms in mothers who experienced greater pandemic-related stress during gestation.

First, this effect seems to be independent of the actual exposure of women to the SARS-CoV-2 virus. In the present sample, we excluded women who tested positive for the COVID-19 by PCR assessment during pregnancy or at delivery. Moreover, we asked women to report any symptoms that could be reminiscent of COVID-19 disease as well as the presence of family members or significant others who were positive, had been hospitalized, or died with COVID-19 when they were pregnant. As we compared women with or without any direct or indirect exposure to the SARS-CoV-2, no significant differences emerged for prenatal pandemic-related stress nor anxious symptoms assessed postnatally. As such, it is plausible to speculate that direct or indirect exposure to COVID-19 did not increase the risk of mental health problems in women during the COVID-19 pandemic. This is noteworthy for clinical practice, as healthcare professionals should not consider the presence of COVID-19 diagnosis as a risk factor for pandemic-related mental health risk and a broader preventive approach should be adopted. Indeed, pregnancy is a period of heightened neuroplasticity for women (1) and this may increase future mothers’ susceptibility to stressful exposures (65). From this perspective, policymakers and healthcare professionals should be aware that pregnant women may be a specific at-risk population during a global pandemic, as they might be exposed to high levels of stress and risk of anxious symptomatology independently from the actual positivity to the virus.

Second, prenatal pandemic-related stress emerged as significantly associated with post-natal anxious symptoms in this sample. This finding confirms previous literature that already demonstrated how high levels of prenatal stress might predispose women to mental health problems after delivery (23). Nonetheless, the percentage of mothers reporting elevations in the standardized anxiety scale was well above (32%) previous reports on similar community samples (i.e., 10–17%) (20, 21). As such, during the present healthcare emergency, this heightened risk for impaired mental health for mothers should not be underestimated. The mental health risk connected to pandemic-related stress may act as a silent pandemic that is relatively independent of SARS-CoV-2 direct or indirect exposure and that may have critical consequences for mothers’ wellbeing. Moreover, previous research has shown that high levels of maternal anxiety after delivery may be a trigger condition for further negative health consequences for mothers as well as for their infants (66). For example, evidence from the Generation R study suggested that mothers with high levels of postnatal anxiety had a higher probability to have infants with difficult temperament characterized by increased motor activity and negative emotionality (67). More recently, regulatory problems have been identified in infants of mothers with elevated levels of postnatal anxiety (68). Moreover, infants of mothers with high levels of anxiety may also develop socio-cognitive problems, such as attention bias toward threat-related stimuli (69). Recognizing, targeting, and taking care of pandemic-related stress with appropriate preventive and dedicated healthcare strategies should be a priority goal of policymakers and clinicians during the time of pandemic we are living in order to promote better maternal health and to prevent long-term detrimental consequences for children development.

Third, higher levels of CpG-specific *BDNF* methylation in the promoter region were found to be significantly associated with both prenatal pandemic-related stress (27% of assessed CpG sites) and maternal post-delivery anxious symptoms (36% of assessed CpG sites). The *BDNF* gene is well-known to be involved in neuroplasticity processes that occur during pregnancy and that constitutes part of the biological communications occurring between the maternal and the fetal compartments (43). Not surprisingly, the *BDNF* regulation has been previously found to be susceptible to stressful exposures occurring during pregnancy (45, 47). It has also been shown that the epigenetic regulation of the *BDNF* gene may be involved in setting the risk for psychiatric and affective disorders, such as depression and anxiety (36, 53). In our sample, a specific CpG site (CpG6; chr11-27,723,190–27,723,191) emerged as a significant mediator of the relationship between pandemic-related stress and post-partum maternal anxiety. This CpG site is included in one of the promoter regions of the *BDNF* gene previously highlighted by Kertes et al. (46) to be plausible loci of epigenetic regulation in relation to both maternal adversity exposure and postnatal mental health issues, including anxiety-related outcomes. This finding suggests that epigenetic regulation of *BDNF* gene by adverse events occurring during pregnancy may play a causal role in contributing to increased risk of maternal anxious symptoms after delivery.

Of course, it should be highlighted that *BDNF* is only one of the genes involved in the risk of affective symptomatology in pregnant women and mothers. Previous research reported on different stress-related genes, including—among others—*BDNF*, but also *SLC6A4* (35, 70), *NR3C1* (33, 71), and *FKBP5* (72). It is possible that the epigenetic regulation occurring at multiple target sites may interact in producing higher rates of mental health risk in pregnant women and mothers and this should be tested in future studies with larger samples that will eventually provide the opportunity to conduct epigenome-wide assessments. Although previous research suggests a global trend in similar methylation levels of *BDNF* assessed in central and peripheral tissues (37), it should be recognized that the actual expression of *BDNF* variants may be under the control of a large number of splices and thus tissue-related differences cannot be excluded (40). We further recognize additional limitations of this study. Although the MOM-COPE is a longitudinal project, the data reported in the present study are cross-sectional and this limits the possibility to draw valid conclusions about the causal directions of associations. We assessed pandemic-related stress with an *ad hoc* self-report questionnaire that was developed to be sensitive to the specific nature of this unprecedented healthcare emergency. The obtained measure is retrospective. Although this may limit the generalizability of these findings, the concordance of our results with previous studies on the association of prenatal stress and maternal anxiety may indirectly corroborate the goodness of our *ad hoc* tool. The Pearson’s bivariate correlation indexes reported are significant, but the strength of the association of *BDNF* CpG-specific% methylation with pandemic-related stress and anxiety symptoms is mild. As such, it is largely possible that other factors may be involved; notably, we did not include women who tested positive for SARS-CoV-2 during pregnancy and we did not collect information on previous stressful or traumatic events that occurred during women’s life. As such, we cannot exclude that additional previous adverse experiences may have already contributed to increased stress susceptibility in these women. In our study, only a specific *BDNF* CpG site was significantly associated with both prenatal pandemic-related stress and postnatal maternal anxious symptoms and was therefore investigated as a mediator. The biological relevance and plausibility of a single CpG site may be controversial; still, it might inform future research questions on the epigenetic mechanisms involved in short- and long-term impact of pandemic-related stress in vulnerable populations.

## Conclusion

In sum, this study provides for the first time evidence of the role played by epigenetic regulation of the *BDNF*—a target gene that has known implications in prenatal stress and psychiatric disorders—in setting the risk of less-than-optimal mental health in pregnant women during the COVID-19 pandemic. These findings have specific implications for scientific advances as well as for healthcare professionals and policymakers. First, the present study contributes to the emerging literature on the behavioral epigenetic vestiges of prenatal stress exposure suggesting that a potential mediation pathway involving increased methylation of the *BDNF* gene may be involved in setting the stage for heightened maternal anxiety soon after delivery. Second, with these findings, we highlight the presence of a hidden and silent pandemic that is relatively independent of the actual positivity to the SARS-CoV-2 virus, but that is likewise risky for mothers’ mental health. Investing in appropriate and timely care solutions for pregnant women and mothers during a time of pandemic should be a priority for perinatal healthcare professionals. Policymakers have the opportunity to strengthen existing services and to promote the development of new actions that prioritize the promotion of health and wellbeing in pregnant women and mothers during the present healthcare emergency.

## Data Availability Statement

The raw data supporting the conclusions of this article will be made available by the authors, without undue reservation.

## Ethics Statement

The studies involving human participants were reviewed and approved by the Ethics committee Pavia. The patients/participants provided their written informed consent to participate in this study.

## Author Contributions

LP and RB: conceptualization. LP and RGio: methodology. LP, RGio, MV, FM, and AC: formal analysis. GB, LD, BG, RGia, MM, RN, CP, FP, EB, and BS: data collection. MV, FM, AC, and SG: data curation. LP and SG: writing—original draft preparation. RGio, MV, FM, and AC: visualization. SO and RB: supervision. LP: project administration and funding acquisition. All authors have read and agreed to the published version of the manuscript.

## Conflict of Interest

The authors declare that the research was conducted in the absence of any commercial or financial relationships that could be construed as a potential conflict of interest.

## Publisher’s Note

All claims expressed in this article are solely those of the authors and do not necessarily represent those of their affiliated organizations, or those of the publisher, the editors and the reviewers. Any product that may be evaluated in this article, or claim that may be made by its manufacturer, is not guaranteed or endorsed by the publisher.
